# Exploring the associations between transcript levels and fluxes in constraint-based models of metabolism

**DOI:** 10.1186/s12859-021-04488-8

**Published:** 2021-11-29

**Authors:** Neeraj Sinha, Evert M. van Schothorst, Guido J. E. J. Hooiveld, Jaap Keijer, Vitor A. P. Martins dos Santos, Maria Suarez-Diez

**Affiliations:** 1 Nutrition, Metabolism and Genomics Group, Division of Human Nutrition and Health, Wageningen University & Research, Stippeneng 4, 6708 WE Wageningen, The Netherlands; 2 Human and Animal Physiology, Wageningen University & Research, De Elst 1, 6708 WD Wageningen, The Netherlands; 3 Laboratory of Systems and Synthetic Biology, Wageningen University & Research, Stippeneng 4, 6708 WE Wageningen, The Netherlands; 4 LifeGlimmer GmbH., Markelstrasse 38, 12163 Berlin, Germany; 5 Bioprocess Engineering Group, Wageningen University & Research, PO Box 16, 6700 AA Wageningen, The Netherlands

**Keywords:** E-Flux, Gene expression integration, Transcriptomics, Constraint-based models, Proportionality constant

## Abstract

**Background:**

Several computational methods have been developed that integrate transcriptomics data with genome-scale metabolic reconstructions to increase accuracy of inferences of intracellular metabolic flux distributions. Even though existing methods use transcript abundances as a proxy for enzyme activity, each method uses a different hypothesis and assumptions. Most methods implicitly assume a proportionality between transcript levels and flux through the corresponding function, although these proportionality constant(s) are often not explicitly mentioned nor discussed in any of the published methods. E-Flux is one such method and, in this algorithm, flux bounds are related to expression data, so that reactions associated with highly expressed genes are allowed to carry higher flux values.

**Results:**

Here, we extended E-Flux and systematically evaluated the impact of an assumed proportionality constant on model predictions. We used data from published experiments with *Escherichia coli* and *Saccharomyces cerevisiae* and we compared the predictions of the algorithm to measured extracellular and intracellular fluxes.

**Conclusion:**

We showed that detailed modelling using a proportionality constant can greatly impact the outcome of the analysis. This increases accuracy and allows for extraction of better physiological information.

**Supplementary Information:**

The online version contains supplementary material available at 10.1186/s12859-021-04488-8.

## Background

Numerous modelling approaches have been developed to describe and investigate the metabolic behaviour of an organism or a living cell [1, 2]. Constraint-based modelling has become one of the most successful and widely adopted approaches for modelling cellular metabolic networks [3, 4]. This approach relies on mass balance over intracellular metabolites and the assumption of pseudo-steady-state conditions to determine intracellular metabolic fluxes. The information about the possible biochemical conversion, transport and uptake or secretion of metabolites is contained in the stoichiometric matrix. An additional set of constraints describe experimental measurements, such as measured uptake rates, reaction reversibility and maximum enzyme capacity. In addition, constraint-based genome-scale metabolic models (GEMs) contain the associations between genes and the corresponding reactions through the so-called gene-protein-reaction (GPR) relationships, expressed through logical (or Boolean) functions [5, 6].

Such stoichiometric models result in an under-determined linear equation system, which is not enough to calculate a unique flux distribution. These models are therefore combined with additional experimental data or assumptions to yield well-defined flux distributions. The assumption of optimality is often used to construct a Flux Balance Analysis (FBA) problem [7]. Under this assumption, FBA is used to find the optimal (maximum or minimum) value of a selected function, called the objective function, compatible with the constraints. Objective functions are often chosen to represent maximization of growth, ATP production, or minimization of glucose consumption among others [8].

One of the limitations of constraint-based modelling is that intracellular fluxes are most often left unconstrained due to lack of knowledge of real flux bounds of the corresponding reactions. Modelling conditions under which the cell behaves optimally, such as exponential growth, allows to side step this limitation by implicitly assuming that cells are able to adjust their metabolism to accommodate the optimal metabolic state. Moreover, these models are not able to account for most of the regulatory activity inside the cells: transcriptional, translational and post-translational regulation. To overcome this limitation different approaches to integrate gene expression data into a metabolic model have been developed [9]. Transcriptomic data provide complete information of regulatory rules which can improve the predictive power metabolic flux distributions in a wide range of states in GEM.

Several algorithms have been developed that demonstrated how gene expression data can be incorporated into metabolic models. These methods further constrain the solution space of the GEM by incorporating expression values as a proxy for flux using different approaches. For instance, iMAT and GIMME assume that mRNA levels below a certain threshold reveal that corresponding reactions are inactive [10, 11]. E-Flux and PROM assume that transcript level indicates the degree to which the reactions are active by constraining the upper bounds [12, 13]. The main assumptions and characteristics of the methods have been reviewed in [14], to where we refer the reader. Nevertheless, a systematic evaluation of their performance shows that there is still a lack of an optimal and general approach [14]. Although methods have been developed to incorporate quantitative proteomics data and enzyme kinetic data to constrain fluxes [15], quantitative proteomics datasets remain hard to come by.

One of the main challenges these methods face is how to link transcript levels, protein levels, enzyme activity and flux values. This is reflected in previous studies where correlations have often been found to be poor in the following cases: (i) between mRNA (gene expression) and protein concentration (abundances) across all genes and proteins expressed in an organism; and (ii) between enzyme activity and metabolic flux, considering combined measurements of gene expression, protein levels and metabolic fluxes [16–19]. This could be due to multiple factors. For instance, enzymes might accept several different substrates thereby participating in multiple reactions thus relating the expression of one gene to several fluxes. For reactions catalysed by enzyme complexes, the opposite situation applies where several genes are related to one flux. Similarly, for isoenzymes different genes are coupled to one or several fluxes depending on interpretation of the inter-conversions of the different enzymes. Finally, there can be instances of combinations of the above cases where many genes are related to many fluxes. Therefore, it is not trivial to make quantitative or even qualitative comparisons between gene expression and metabolic flux. Methods to integrate gene expression data and metabolic models assume, in most cases, that the structure of the network combined with the expression data, retains enough information about the state of the system to lead to meaningful predictions [20].

E-Flux is an algorithm that relates flux bounds with gene expression data so that reactions associated with highly expressed genes are allowed to carry higher flux values [12]. This method does not assume that enzyme concentrations, activities or kinetics are the only determinants of reaction fluxes. E-Flux constrains the upper bound of a reaction according to the expression of the associated genes relative to a specific threshold. In cases where the gene expression level is below a certain threshold, tight constraints are placed on the flux through the corresponding reactions. The rationale behind E-Flux is that mRNA levels can be used as an approximation to the amounts of protein available, and these in turn can be used as an approximation to the upper bound on reaction rates. The E-Flux algorithm was originally developed for global microarray data and was later adapted to RNAseq data [20]. In these calculations normalized gene expression is used to constrain the fluxes. Thus, a proportionality constant (PC) is implicitly included that models the gene specific link between expression and flux. Implicit inclusion means that these factors are often taken to have a unit value. The PC is unique to each reaction in GEM and would thus implicitly account for a broad range of effects, like translation efficiency, protein degradation rate and enzyme kinetics. The value of this PC greatly impacts the results of the metabolic simulations. A too high value would result in reaction upper bound so high that effectively it does not constrain the reactions. A too low value would over-constrain the model, effectively preventing reactions from carrying any flux and leading to an infeasible model, as constraints associated for instance with maintenance requirements or thermodynamic requirements on reaction reversibility cannot be fulfilled.

Here, we present a systematic evaluation of the impact of various PC on the performance of E-Flux algorithm. To this end, we have selected published data from two studies in *Escherichia coli* and one in *Saccharomyces cerevisiae* that have been used earlier for systematic evaluation of methods for data and model integration [14]. The value of this PC can greatly influence the accuracy of the predictions and our result shows that a consistent choice can greatly increase the model’s predictive power. In addition, we provide suggestions for selection of optimal values. The presented approach is a novel extension of the E-Flux algorithm, is generic and can be adapted to other methods for data and model integration.

## Results

In order to evaluate the impact of the PC after integration of transcriptomics data, we have selected four studies: Ishii et al*.* [21], Holm et al*.* [22] and Gerosa et al*.* [23] for *E. coli*; and Rintala et al*.* [24] for *S. cerevisiae.* Three of these studies have previously been selected as a gold standard for assessment of methods to integrate expression data and metabolic models [14, 21, 22, 24]. The Gerosa et al*.* dataset complements those studies as it also provides intracellular flux measurements.

For the selected datasets and models, we have applied the E-Flux algorithm with varying values of the PC and used the integrated model to predict a selected phenotype measurement (here the growth rate). We observed that the value of the PC highly impacts the growth rate prediction and we have fitted the value for the PC to that producing best agreement between model prediction and measured value. This PC value was then used to predict additional phenotypes (secretion rates and/or flux through intracellular reactions).

### *E. coli* (Holm et al.)

**Fig. 1:**
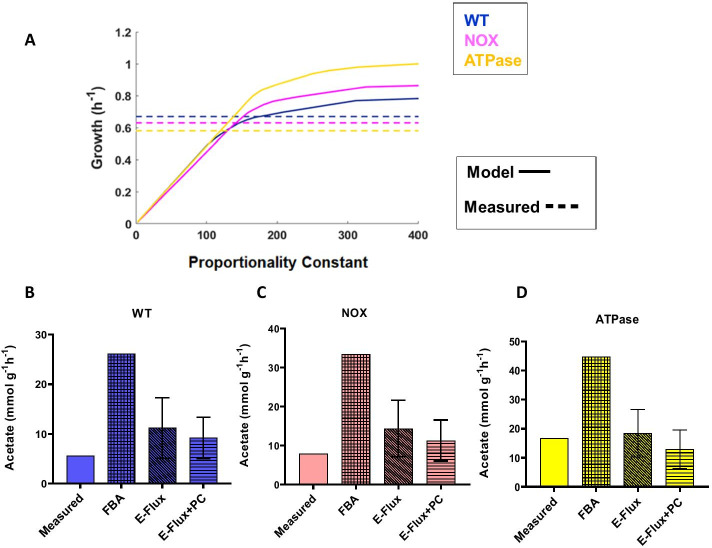
Exploration of impact of proportionality constant on predicted flux in WT and two mutant strains overexpressing NOX and ATPase, respectively, in *E. coli* (Holm dataset): **A** growth, **B** WT acetate secretion, **C** NOX acetate secretion, **D** ATPase acetate secretion. Data from growth simulations in (**A**) are used to identify optimal values for the proportionality constant as those for which model predictions match experimental values (119.8, 141.90 and 175.4 for ATPase, NOX and WT strains respectively). Simulations of acetate production (**B**, **C**, **D**), are done by fixing the fitted values followed by sampling of the solution space. The error bars indicate the standard deviations

This study reports *E. coli* strains growing aerobically in batch culture [22]. The considered strains are wild-type (WT) MG1655 and two mutant strains over-expressing NADH: flavin oxidoreductase/NADH oxidase (NOX) and *atpAGD* (F1-ATPase), respectively. A major impact of the introduced genetic mutations is the reduction in growth rates (shown in Fig. 1A), even when there is a major increase in glucose uptake rate: 27% and 70% for the NOX and ATPase mutant respectively [22]. We have used gene expression data from this study to constrain the *E. coli* GEM. GEMs are often used to predict growth rates from carbon uptake rates. The nature of these GEM and the optimality principle in FBA ensures that (in the absence of additional constraints) higher uptake rates correspond to higher growth rate predictions. However, the integration of expression data and explicit inclusion of a proportionality constant influences this behaviour as shown in Fig. 1A. As previously stated, large values of the proportionality constant lead to reaction bounds so high that they effectively do not constraint the reactions. This is clearly seen in Fig. 1A, where for large values of the PC (> 150) growth rate predictions were higher for the mutant strains NOX and ATPase than for WT, following the glucose uptake measurements.

However, lower PC values (< 100) do show the reduced growth rate of the mutant as compared to the wild type. Comparison of the model predictions and measured growth rates lead us to select, for each strain, the PC that provides the best match. These values were found to be 119.8, 141.90 and 175.4 for ATPase, NOX and WT strains respectively.

Once these fitted values were included, the model was used to predict acetate secretion by sampling the steady-state flux space. Predictions for acetate secretion show the measured trend, with the lowest secretion rate in WT (9.15 ± 4.71 mmol g^−1^ h^−1^) Fig. 1B followed by mutant strain NOX (11.34 ± 5.25 mmol g^−1^ h^−1^) Fig. 1C and the highest secretion in mutant strain ATPase (12.89 ± 6.61 mmol g^−1^ h^−1^) Fig. 1D. For WT and NOX the predicted flux overestimates the measured one, while for the ATPase the predicted flux was lower than the measured flux. In two of the three cases inclusion of the PC improves the predictions.

### *E. coli* (Ishii et al.)

In their work, Ishii and co-workers experimentally investigated the response of *E. coli* to environmental and genetic perturbations and provided multiple high-throughput omics data for both wild-type and mutant strains. To study the effect of environmental perturbations, they cultured WT cells at various dilution rates, while the effects of genetic perturbations were examined by disrupting 24 single genes in the glycolysis and in the pentose phosphate pathways. In order to understand how phenotype modelling predictions are improved upon integration of experimental data. We have used gene expression data from *E. coli* strains growing aerobically in a chemostat at a higher dilution rate of 0.7 h^−1^ [21].

**Fig. 2:**
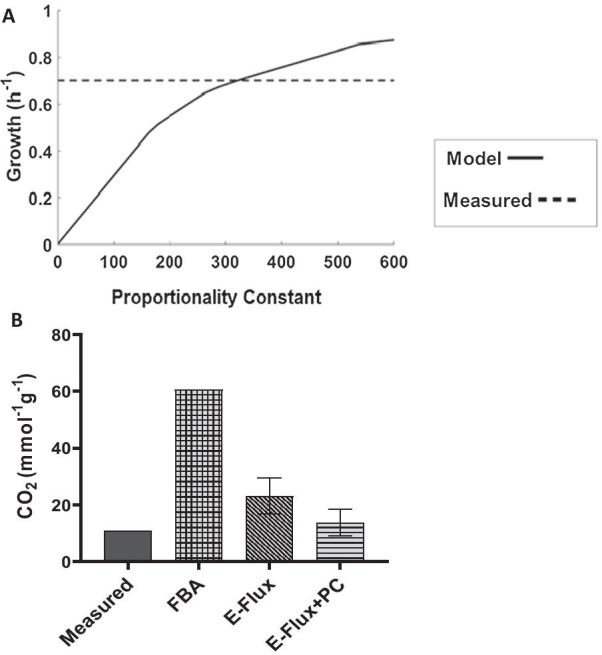
Exploration of proportionality constant on Predicted flux in *E. coli* for the Ishii dataset: **A** growth, **B** CO_2_ secretion**.** Data from growth simulations (**A**) are used to identify optimal values for the proportionality constant as those for which model predictions match experimental values of 322.80. Simulations of CO_2_ production (**B**) is done by fixing the fitted values followed by sampling of the solution space. The error bars indicate the standard deviations

First, we used the measured growth rate to estimate the value of the PC, so that model predictions best fit the data. This fitting led to a PC of 322.80, as shown in Fig. 2A. Once the PC was fit, we used the parametrized model to predict CO_2_ secretion rates. This showed that secretion rates were slightly overestimated by the model when compared to measured secretion rates. In this case the predicted secretion rate is 13.8 ± 4.67 mmol g^−1^ h^−1^ (Fig. 2B), while the measured secretion rate was 10.83 mmol g^−1^ h^−1^. Similarly, we used the model to predict production rates for other fermentation products such as acetate, ethanol, lactate, pyruvate and succinate for which no secretion was predicted regardless of the inclusion of the PC (Additional file 4: Figure S1). In the case of pyruvate, ethanol and acetate this contrasted with the experimental measures.

### *E. coli* (Gerosa et al.)

**Fig. 3:**
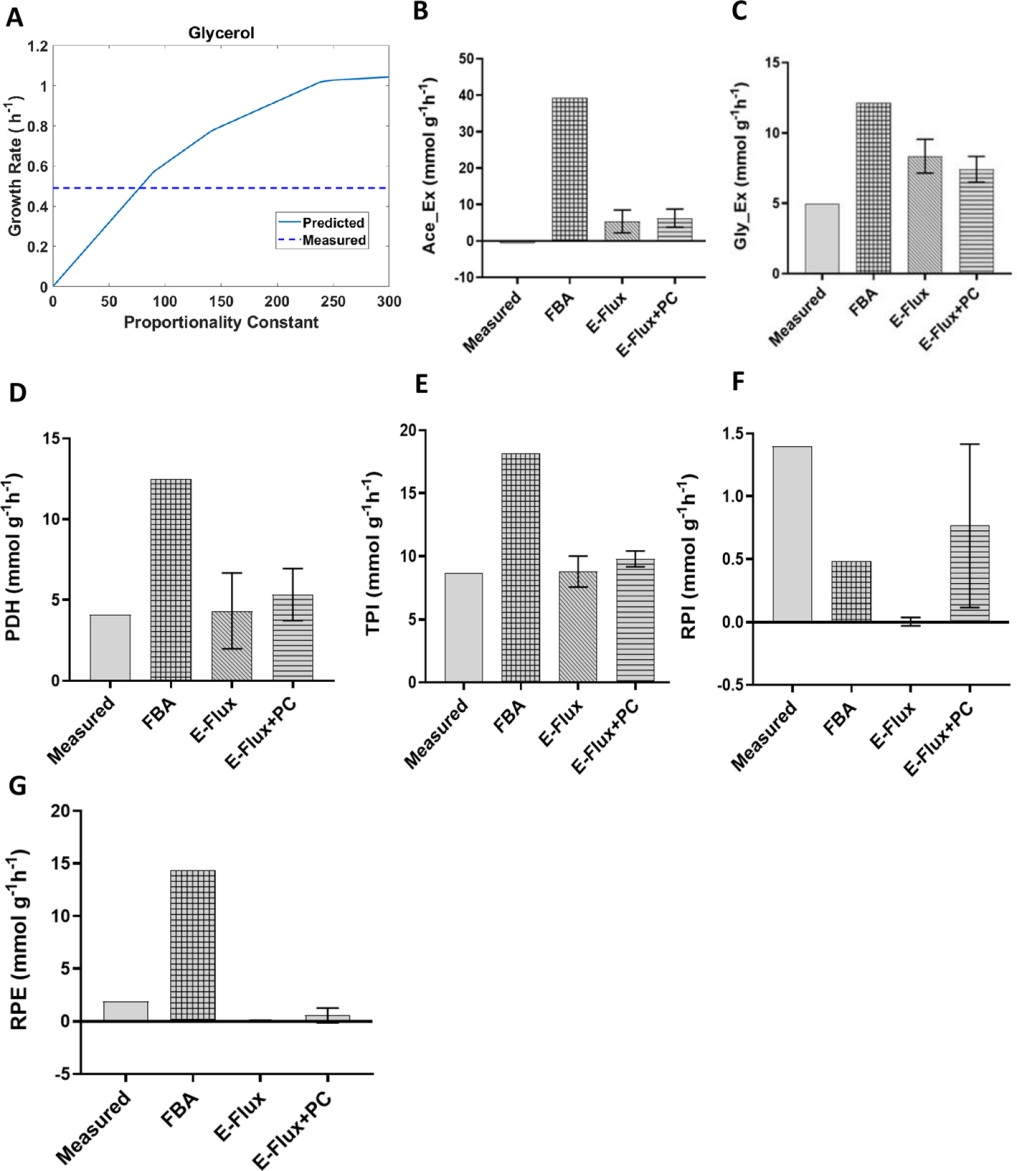
Exploration of impact of proportionality constant on predicted flux in glycerol carbon source in *E.coli* (Gerosa dataset): **A** growth, **B** acetate uptake/ = secretion, **C** glycerol uptake, **D** pyruvate dehydrogenase (PDH), **E** triose-phosphate isomerase (TPI), **F** ribose-5-phosphate isomerase (RPI), **G** ribulose 5-phosphate 3-epimerase (RPE). Data from growth simulations (**A**) are used to identify optimal values for the proportionality constant as those for which model predictions match experimental values. Simulations of internal and external flux were done by fixing the fitted values followed by sampling of the solution space. The error bars indicate the standard deviations

In this study, Gerosa et al*.* developed an experimental-computational approach to decipher the regulatory events that drive cellular adaptations between carbon sources in *E.coli* [23] Thereby generating data on metabolite concentrations, transcript levels, and ^13^C-tracer data during exponential growth. This dataset was used to evaluate performance when predicting intracellular fluxes. We first used the measured growth rate to estimate the PC in glycerol as a carbon source which corresponds to a value of 76.32 (Fig. 3A). This could suggest that the metabolic network has adapted to growth in glycerol carbon source without further constraining the model with gene expression data. Once the PC was fit the model was used to predict all possible fluxes using E-Flux + PC by sampling the steady-state flux space. Figures 3B-G show predictions for the internal and external fluxes for a selected set of reactions. Simulation predicted no secretion rates for fructose, galactose and gluconate under this condition (Additional file 1 and Additional file 5: Figure S2). This was comparable to the result shown by Gerosa et al. [23].

The results for the different carbon sources considered are summarized on Table 1 (see full results in Additional file 1). For the fluxes considered in this data set, the introduction of the proportionality constant improves flux predictions in about 70% of the cases.

**Table 1:** Comparison between E-Flux + PC and E-Flux on predicted intra- and extra-cellular fluxes of *E. coli* growing on different carbon sources (Gerosa et al.)

Carbon Source	All measured reactions		Uptake /secretion	
	E-Flux + PC	E-Flux	E-Flux + PC	E-Flux
Glycerol	15	6	8	2
Glucose	13	8	8	2
Acetate	14	7	6	4
Pyruvate	11	10	7	3
Gluconate	14	8	7	3
Succinate	17	4	8	2
Galactose	14	7	8	2
Fructose	18	3	8	2

The table indicates the number of reactions for which each algorithm predicts values more similar to the measured ones. Full results are provided in Additional file 1

### *S. cerevisiae* (Rintala et al.)

**Fig. 4:**
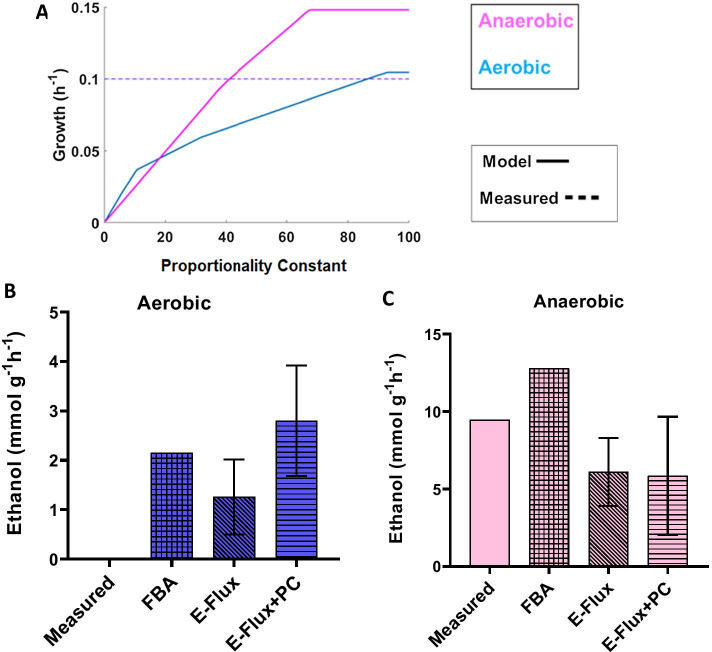
Exploration of impact of proportionality constant on predicted flux in anaerobic and aerobic in *S. cerevisiae* (Rintala dataset): **A** growth, **B** aerobic ethanol secretion, **C** anaerobic ethanol secretion. Data from growth simulations (**A**) are used to identify optimal values for the proportionality constant as those for which model predictions match experimental values (40.6 and 86.56 for anaerobic and aerobic strains respectively). Simulations of ethanol production (**B** and **C**) are done by fixing the fitted values followed by sampling of the solution space. The error bars indicate the standard deviations

Rintala et al*.* [24] grew *S. cerevisiae* strains in a glucose-limited chemostat with a dilution rate of 0.1 h^−1^ at different oxygen levels. These include intermediate oxygen levels, ranging from fully anaerobic to fully aerobic. The dataset contains genome-wide gene expression data from microarray. Fluxomic data for the same conditions were obtained from Jouhten et al*.* [25]. We analysed growth and ethanol production in two extreme conditions: fully aerobic as well as anaerobic growth, as shown in Fig. 4. Again, growth data were used to establish the optimal values for the PC in aerobic and anaerobic conditions. These values correspond to 40.06 for anaerobic growth and 86.56 for aerobic growth as shown in Fig. 4A. This could suggest that the metabolic network of *S. cerevisiae* is adapted to aerobic growth and no further regulation of gene expression is needed for these conditions. As previously stated, high values of the PC lead to flux boundaries so high that they do not effectively impact model predictions. Under aerobic conditions this would correspond to values of 96 or higher.

Since *S. cerevisiae* is commonly employed to produce ethanol, we used FBA to calculate production of ethanol by fixing the PC obtained from growth in aerobic and anaerobic conditions. In both cases, we still assume maximum growth rate and sampling of the solution space is performed to identify ethanol production rates compatible with maximum growth. Aerobic ethanol simulations predicted by the model showed flux values of 2.8 ± 1.12 mmol g^−1^ h^−1^, whereas the measured production rate was zero Fig. 4B. From the results shown in Fig. 4C, for the anaerobic condition E-Flux predicted a flux value of 5.86 ± 3.81 mmol g^−1^ h^−1^ which is lower than the measured ethanol production at a rate of 9.46 mmol g^−1^ h^−1^. Similarly, we used the model to predict production rates for other products, such as glycerol and acetate. We observed that there were no predicted production rates for acetate in both aerobic and anaerobic conditions. This was comparable to the experimental values reported by Rintala et al. In the case of glycerol, there were no predicted production rates in aerobic and anaerobic conditions. Although in agreement with the measured production rates in the aerobic conditions, this was not the case for anaerobic conditions (measured value = 1.094 mmol g^−1^ h^−1^; predicted value = 0 mmol g^−1^ h^−1^). Similarly, no secretion rates were predicted for acetate when E-Flux + PC algorithm was applied to the Rintala dataset (Additional file 6: Figure S3). This is supported by the fact that no predicted secretion rates were observed for any of the metabolites if the E-Flux algorithm without any modification was used. In summary, in five out of the six considered rates, inclusion of the PC does not modify the E-Flux predicted values. Only in one case, ethanol secretion in aerobic conditions, the results are changed, and in that case both algorithms wrongly predict secretion.

## Discussion

Gene expression is known to play a major role in controlling metabolism when there is a significant change in gene expression between different conditions. Several studies in the past show a strong qualitative relation between gene expression and metabolic flux, especially in the case of microbes [26, 27]. Association between transcript levels and reaction fluxes could be represented through a reaction specific proportionality constant that would capture and, to some extent, summarize transcription, translation and degradation dynamics as well as reaction kinetics. Detailed knowledge of these constants for every reaction in the model would entail exhaustive knowledge and measurements not currently available. Therefore, and for practical purposes, we have considered a single PC for all gene/reaction pairs and we have considered it as a pure phenomenological constant that provides an extra degree of freedom when reproducing a systems level measurement. Availability of additional datasets and detailed reaction information would allow, to some extent, estimation of reaction specific constants or value ranges that could be used for ensemble modelling. For this task, Bayesian statistical learning shows promise as demonstrated by Li et al*.* [28]. We can envisage a set up on which for each reaction a precise determination of the link between transcript level and activity is established by measuring transcript levels, protein levels and reaction kinetics. This approach is used when building dynamic models of metabolism based on differential equations of a subset of relevant reactions. However, this is a data intensive approach that can only be applied in a reduced set of cases.

In this study we integrate a single measure at systems level (growth rate) with the expression data and show how this can improve predictions without dramatically overfitting the model. Here, we have assessed the impact of using different values of proportionality constant, based on the phenotypic parameter growth, to model proportionality between transcript abundances and fluxes to make accurate predictions on the fluxes. The underlying assumption is that even though the correlation is unknown, the metabolic network retains additional information that led to more accurate predictions.

We have used one set of measurements (growth) for parameter estimation and fitting. The new parametrized model was then used to make predictions on secretion rates for specific metabolites (Fig. 2B); our algorithm predicted secretion rates for CO_2_ were higher than those of the Ishii dataset. However, we did not predict any secretion rate for pyruvate, ethanol, acetate, succinate and lactate (Additional file 4: Additional Figure S1). This is maybe due to the fact that 1) the E-Flux method, as an algorithm, does not incorporate the biological principles that govern the cellular response, and 2) this method is designed for making quantitative predictions and not for qualitative predictions. Therefore, this methodology that introduces a varying proportionality constant gives more insights for correlation of transcriptomics and metabolic flux as compared to existing methods which only consider unit values, such as the original E-Flux method. However, the applicability of these varying proportionality constant in terms of model performance is dependent on many other factors. Apart from the specificity of the algorithm, there could also be other factors affecting the performance of the method. This was previously seen on a study where algorithms such as pFBA, GIMME, iMAT, MADE etc. were tested and some metabolites were wrongly predicted irrespective of the algorithm [14].

Identifying the parameter that most accurately predicts cellular metabolism under a given condition can be viewed as a way to improve FBA calculations, leading to a better understanding of metabolism. Here, we have used growth to fit the proportionality constant, as it is a comprehensive measurement of the status of the organism. In some experimental cases other phenotypes and objective functions could be considered for fitting the proportionality constant. For instance, secretion rates of other metabolites could also be used for the fitting, but it is not very common to do so. Growth as the objective function is considered more suitable for bacterial cells (growth focused), whereas in mammalian cell’s objective functions, such as ATP production, glucose consumption, etc., are likely more reflective of the physiological state of mammalian tissue (maintenance focused). By choosing the objective function most appropriate for the physiological state of a system, this method can potentially be applied to many systems.

## Conclusion

When using expression data to predict metabolic fluxes, a choice of proportionality constant is necessary to link gene expression levels with metabolic fluxes. In any case, a choice has to be made on how these two levels are related to each other. Therefore, simplifications have to be introduced, as it is often not possible to perform a detailed analysis for each and every gene and its reaction(s). The extension of E-Flux presented here used a constant empirically informing on this correlation at the systems level (here growth rate). The results show that in many cases E-Flux + PC performs better and without losing accuracy in the rest of the cases, thus we recommend using the E-Flux + PC approach. Such an approach can be extended to any of the commonly used algorithms, thereby improving their performance. Furthermore, the approach that we have described here is not restricted only to the E-Flux method but also to other algorithms to integrate gene expression data with GEMs.

## Methods

### E-Flux algorithm with proportionality constant

In E-Flux, gene expression data are used to constrain the associated reactions. Gene expression data are initially normalized by dividing them by the maximum level of all the measured genes (g_max_):

1 $${g}_{norm, i}= \left[\left(\frac{1}{{g}_{max}}\right){g}_{i}\right],$$

where ${g}_{i}$ is the expression level of gene *i*. Here, we assume gene expression values have been pre-processed using algorithms suitable to the corresponding technology (global microarray or RNAseq measurements). It should be noted that these algorithms often include a so-called normalization step to eliminate possible technology specific biases (such as those due to different library depth in RNA sequencing experiments). This normalization step should not be confused with the one described below.

To evaluate the impact of differences in the proportionality constant used for scaling gene expression levels and relate them to fluxes, we have explicitly introduced a proportionality constant in the scaling process. Equation 1 is thus replaced by:

2 $${g}_{norm, i}= \left[\left(\frac{1}{{g}_{max}}\right){g}_{i}\times {\delta }_{i}\right].$$

Here, ${\delta }_{i}$ is a gene specific factor that in principle would incorporate effects related to transcription and translation rates, degradation rates and post-translational modifications, among others thereby quantitative linking transcript levels and enzyme activity values. Knowledge of this constant requires detailed knowledge, or at least a close approximation, of how a transcription level relates to an enzyme activity for a specific transcript/protein in the model. This knowledge is not (yet) available and, in the following, we will use a common $\delta$ value for all genes, to which we will refer as PC.

After introducing this constant, the remaining steps of the E-Flux algorithm have been left unmodified. In brief, the scaled gene expression values are used to evaluate the Boolean rules in the GPR associations. In the case of “OR” relationships, describing isozymes, these values are added, in the case of “AND” relationships, describing complex formation, the minimum value in the corresponding set is selected. The so obtained values are then used to adjust reaction bounds in the model. For irreversible (unidirectional) reactions the value is used to set the upper bound. For reversible (bidirectional) reactions, lower and upper bounds are set to ± the value.

To fix the value of the PC, an initial analysis was run on which the impact of the PC on the selected measurement informing on the state of the system (here growth) was evaluated. For this, PC values in the [0, 600] range were taken and for each of them the growth rate was computed. The upper limit of the PC value range was tested to ensure that a sufficiently high value had been explored and that the growth rate prediction had reached a plateau. Then, the PC value was set at the value where the measured value intersected the predicted value.

### Metabolic models

Simulations have been performed using the *E. coli* GEM iAF1260 and the *S. cerevisiae* GEM iTO977 [29, 30]. iAF1260 consists of 1260 metabolic genes, 2077 reactions and 1039 unique metabolites. iTO977 has four compartments, namely cytoplasm, mitochondrion, peroxisome, and extracellular. iTO977 consists of 977 unique genes, 1566 reactions and 1353 metabolites. Models were obtained from supplementary files from previously published studies by Feist et al*.* and Österlund et al*.* [29, 30].

### Data

We obtained data published by Ishii et al*.*, Holm et al*.* and Gerosa et al*.* for *E. coli*, and by Rintala et al*.* for *S. cerevisiae*, where both expression data and ^13^C flux were measured under identical conditions [21–24]. Flux measurements and expression data after pre-processing are given in Additional file 2 and Additional file 3, respectively.

### Model simulations

Maximization of flux through the biomass synthesis reaction was set as objective for the FBA problem in order to simulate growth. For the simulations, constraints related to nutrient uptake (bounds of the corresponding exchange reactions) were modified and experimental values from the respective datasets were used instead. Calculations presented in this manuscript have been performed using MATLAB R2017b (The Mathworks, Inc.) with Gurobi Optimizer 6 (Gurobi Optimization, Inc) and the COBRA Toolbox v2.0[31]. SBMLToolbox was used to convert a SBML (Systems Biology Markup Language) model into a MATLAB data structure [32].

### Sampling the steady state

Sampling the steady-state flux space was performed using the random walk algorithm artificial centering hit-and-run (ACHR) [33]. Sampling the solution space allows us to investigate the flux distributions that satisfy the steady state condition. The ACHR algorithm method chooses an initial point within the solution space. It then calculates warm-up points from the initial point using several iterations of a basic hit-and-run algorithm [34]. These warm-up points are stored as columns of a matrix $W$, and an approximate centre, $s$, is calculated. The direction for the next iteration from a sample point, ${x}_{m}$, is chosen by randomly taking one-point $y$ out of the matrix $W$ and applying the direction vector of y and $s (y\to - s\to )$ to ${x}_{m}$. At each iteration, the newly calculated point, ${x}_{m}+1$, is substituted randomly into $W$ in the place of a previously calculated point [34]. After each iteration, approximate values of the centre are recalculated.

Here, in each sampling procedure, 10,000 randomly distributed points were computed with 200 iterations between each point. All sampling calculations were done in MATLAB version R2017b using the COBRA toolbox v2.0 and Gurobi solver version 6 [3].

## Supplementary Information

**Additional file 1: Figure S1.** Exploration of proportionality constant on predicted flux in *E. coli* for the Ishii dataset: (A) Pyruvate (B) Ethanol (C) Acetate (D) Succinate (E) Lactate

**Additional file 2.** Comparison between measured and predicted flux values using FBA, E-Flux and E-Flux+PC. Data correspond to intra- and extra- cellular fluxes of *E. coli *growing on different carbon sources (Gerosa et al).

**Additional file 3: Figure S2.** Exploration of impact of proportionality constant on predicted flux in glycerol carbon source in *E.coli* (Gerosa dataset): (A) Fumarate secretion (B) Acetate uptake/secretion (C) Fructose uptake (D) Glycerol uptake (E) Glucose uptake (F) Galactose uptake (G) Gluconate uptake (H) Pyruvate uptake (I) Succinate uptake (J) Lactate secretion (K) PGI{Glucose-6-phosphate isomerase} (L) PFK{Phosphofructokinase} (M) FBA{Fructose-bisphosphate aldolase} (N) PDH{ Pyruvate dehydrogenase} (O) TPI{ Triose-phosphate isomerase} (P) RPI{ Ribose-5-phosphate isomerase} (Q) RPE{ Ribulose 5-phosphate 3-epimerase} (R) TKT2{ Transketolase} (S) PPC{ Phosphoenolpyruvate carboxylase} (T) PPCK{ Phosphoenolpyruvate carboxy kinase} (U) FUM{ Fumarate}. Simulations of internal and external flux was done by fixing the fitted values followed by sampling of the solution space. The error bars indicate the standard deviations.

**Additional file 4: Figure S3** Exploration of impact of proportionality constant on predicted flux in anaerobic and aerobic in *S. cerevisiae*: (A) Acetate aerobic (B) Acetate anaerobic (C) Glycerol aerobic (D) Glycerol anaerobic

**Additional file 5.** Flux measurements after pre-processing for the data sets considered in this study.

**Additional file 6.** Expression data after pre-processing for the data sets considered in this study.

## Data Availability

All data generated or analysed during this study are included in this published article (and its Additional files).

## Abbreviations

ACHR: Artificial centering hit-and-run
ATP: Adenosine triphosphate
FBA: Flux balance analysis
GEM: Genome scale metabolic model
GPR: Gene-protein-reaction
NADH: Reduced nicotinamide adenine dinucleotide
PC: Proportionality constant
RNA: Ribonucleic acid
SBML: Systems Biology Markup Language
