# Supplementary material for: Exploring the associations between transcript levels and fluxes in constraint-based models of metabolism
Source: BMC Bioinformatics. 2021 Nov 29;22:574. doi: 10.1186/s12859-021-04488-8 (PMC8628452; doi:10.1186/s12859-021-04488-8)
Supplement: Supplementary file 1 — Additional file 1: Figure S1. Exploration of proportionality constant on predicted flux in E. coli for the Ishii dataset: (A) Pyruvate (B) Ethanol (C) Acetate (D) Succinate (E) Lactate [file 12859_2021_4488_MOESM1_ESM.docx]

**Ishii Data Set**
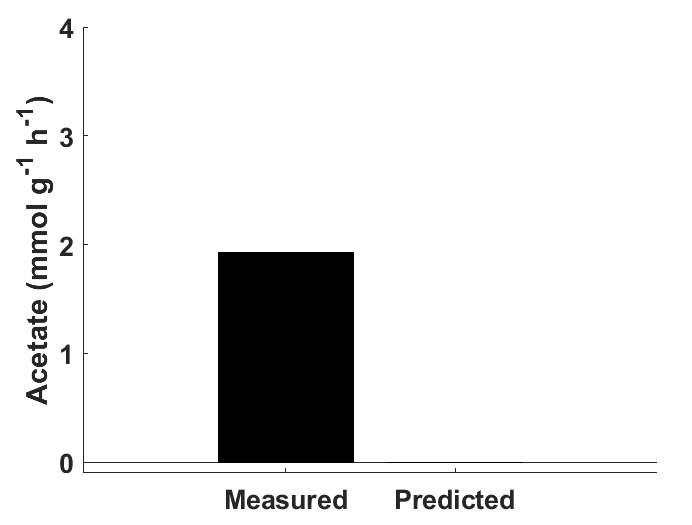


**D**

**C**

**A**

**B**


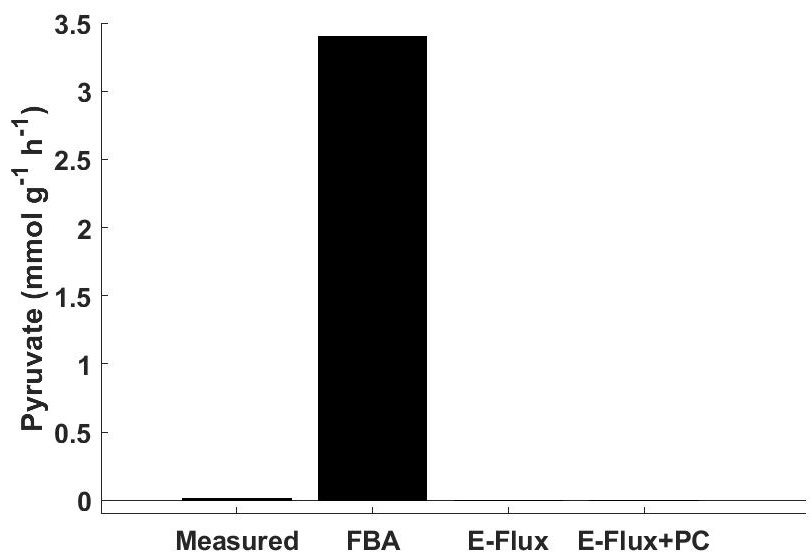

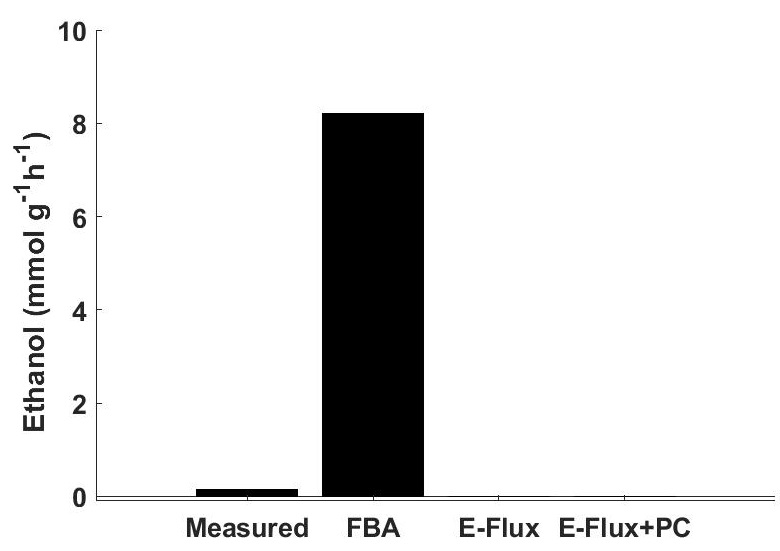


**C**

**D**


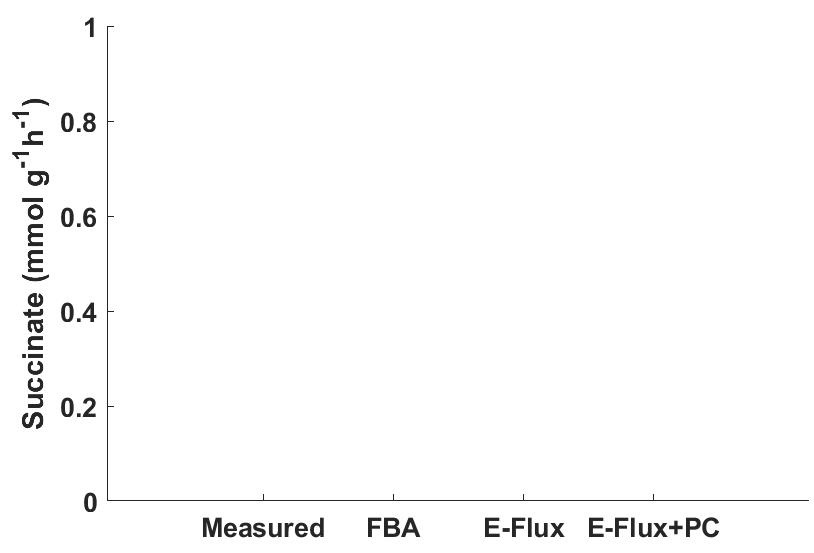

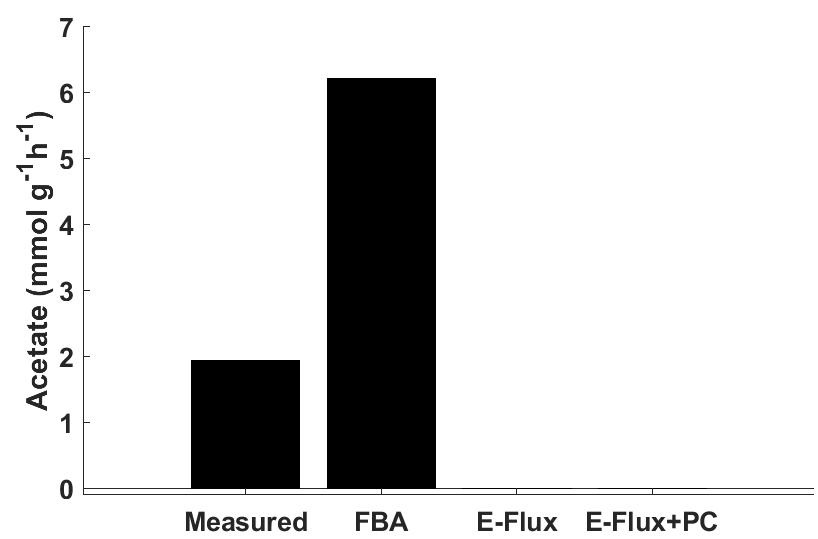


**E**


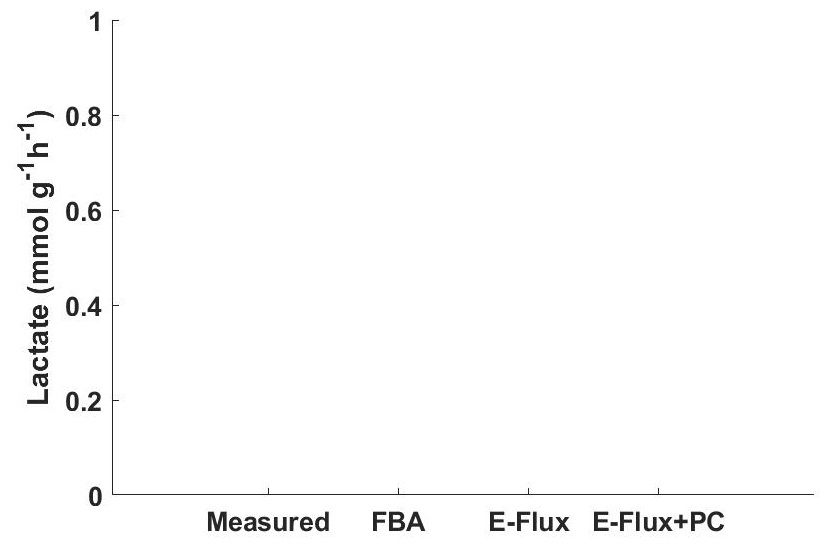
 **Additional Figure 1.** Exploration of proportionality constant on Predicted flux in *E. coli* for the Ishii dataset: (A) Pyruvate (B) Ethanol (C) Acetate (D) Succinate (E) Lactate
