# Supplementary material for: Exploring the associations between transcript levels and fluxes in constraint-based models of metabolism
Source: BMC Bioinformatics. 2021 Nov 29;22:574. doi: 10.1186/s12859-021-04488-8 (PMC8628452; doi:10.1186/s12859-021-04488-8)
Supplement: Supplementary file 3 — Additional file 3: Figure S2. Exploration of impact of proportionality constant on predicted flux in glycerol carbon source in E.coli (Gerosa dataset): (A) Fumarate secretion (B) Acetate uptake/secretion (C) Fructose uptake (D) Glycerol uptake (E) Glucose uptake (F) Galactose uptake (G) Gluconate uptake (H) Pyruvate uptake (I) Succinate uptake (J) Lactate secretion (K) PGI{Glucose-6-phosphate isomerase} (L) PFK{Phosphofructokinase} (M) FBA{Fructose-bisphosphate aldolase} (N) PDH{ Pyruvate dehydrogenase} (O) TPI{ Triose-phosphate isomerase} (P) RPI{ Ribose-5-phosphate isomerase} (Q) RPE{ Ribulose 5-phosphate 3-epimerase} (R) TKT2{ Transketolase} (S) PPC{ Phosphoenolpyruvate carboxylase} (T) PPCK{ Phosphoenolpyruvate carboxy kinase} (U) FUM{ Fumarate}. Simulations of internal and external flux was done by fixing the fitted values followed by sampling of the solution space. The error bars indicate the standard deviations. [file 12859_2021_4488_MOESM3_ESM.docx]

**Gerosa dataset**


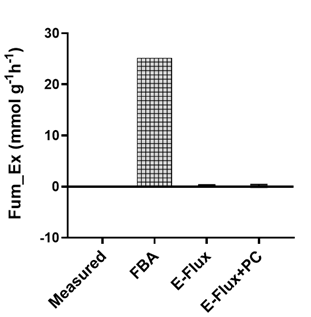

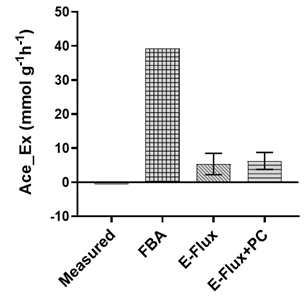

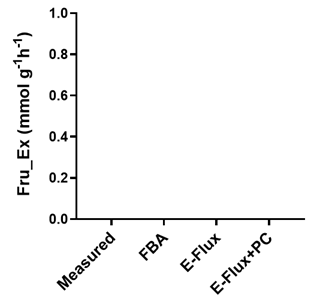

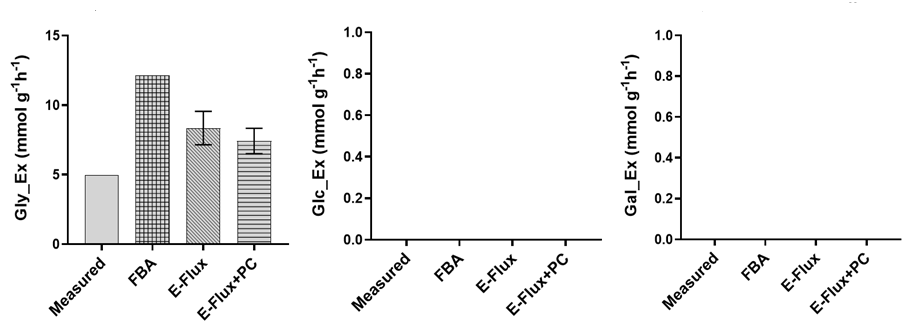


**A**

**B**

**C**

**D**

**E**

**F**


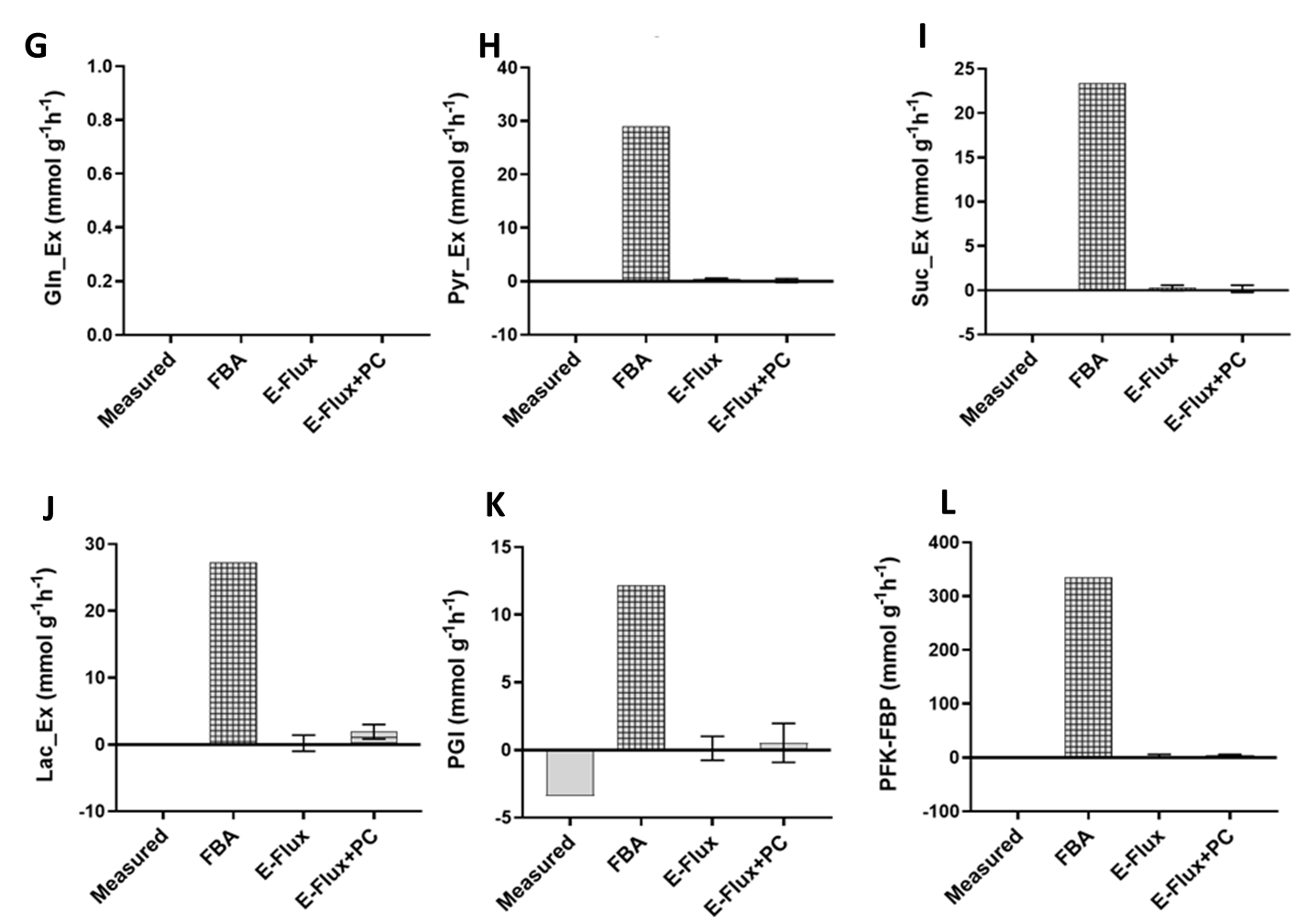


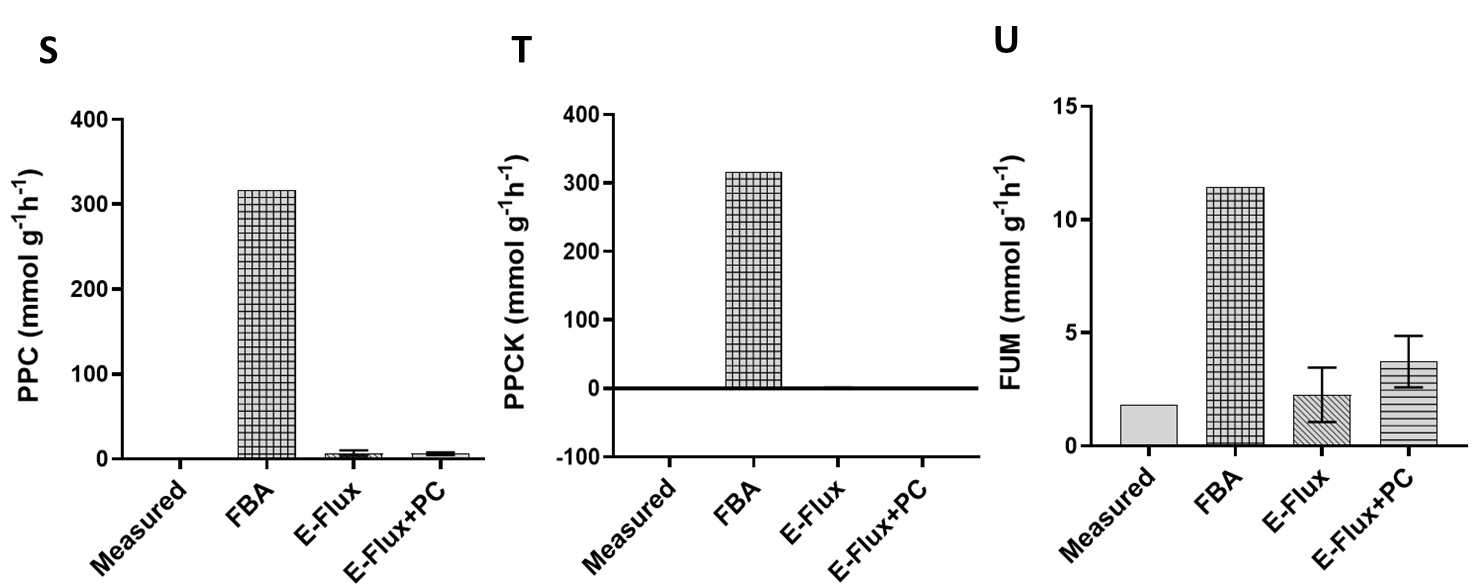

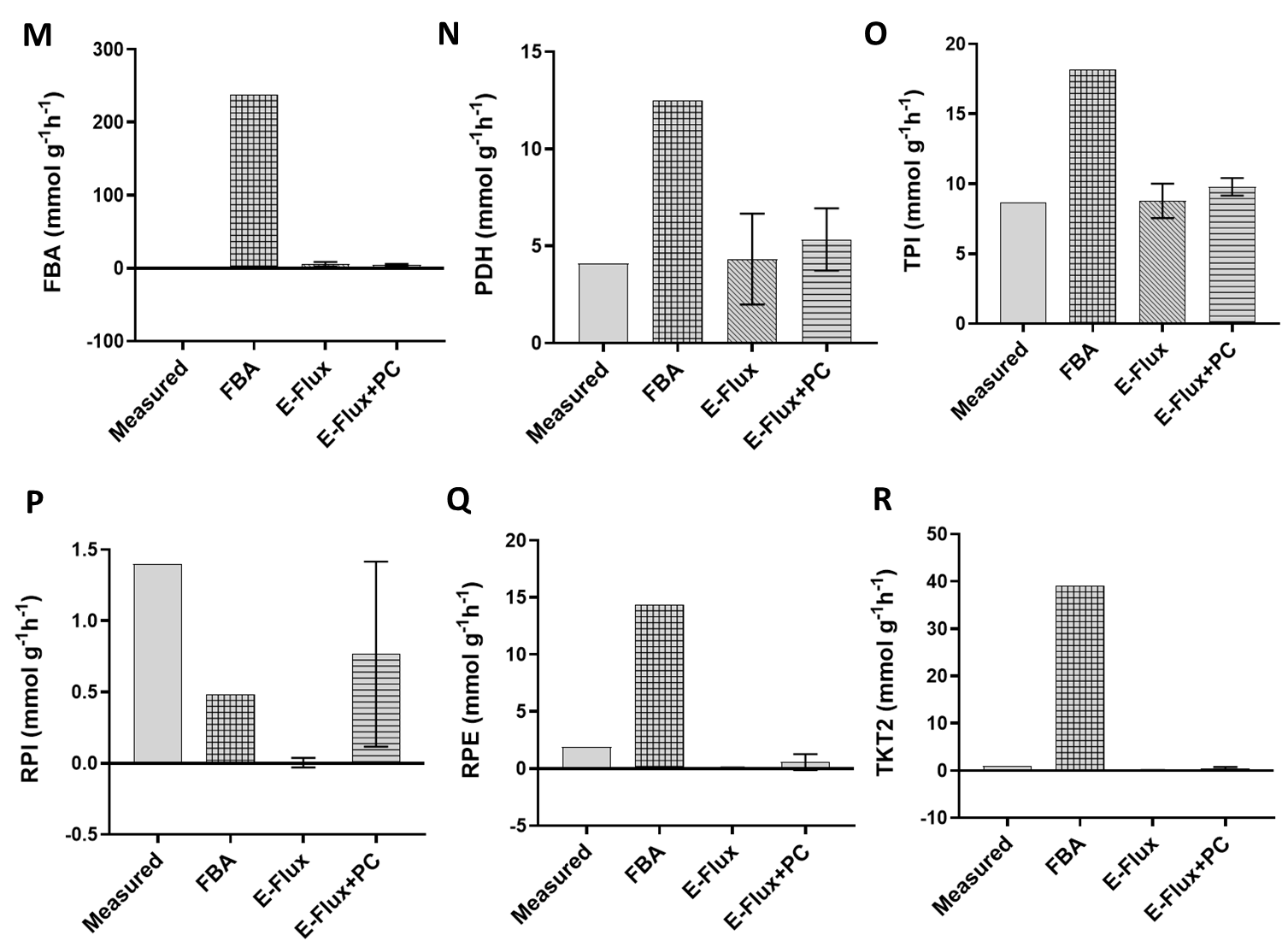


**Additional Figure 2** Exploration of impact of proportionality constant on predicted flux in glycerol carbon source in *E.coli* (Gerosa dataset): (A) Fumarate secretion (B) Acetate uptake/secretion (C) Fructose uptake (D) Glycerol uptake (E) Glucose uptake (F) Galactose uptake (G) Gluconate uptake (H) Pyruvate uptake (I) Succinate uptake (J) Lactate secretion (K) PGI{Glucose-6-phosphate isomerase} (L) PFK{Phosphofructokinase} (M) FBA{Fructose-bisphosphate aldolase} (N) PDH{ Pyruvate dehydrogenase} (O) TPI{ Triose-phosphate isomerase} (P) RPI{ Ribose-5-phosphate isomerase} (Q) RPE{ Ribulose 5-phosphate 3-epimerase} (R) TKT2{ Transketolase} (S) PPC{ Phosphoenolpyruvate carboxylase} (T) PPCK{ Phosphoenolpyruvate carboxy kinase} (U) FUM{ Fumarate}. Simulations of internal and external flux was done by fixing the fitted values followed by sampling of the solution space. The error bars indicate the standard deviations.
