# Supplementary material for: Exploring the associations between transcript levels and fluxes in constraint-based models of metabolism
Source: BMC Bioinformatics. 2021 Nov 29;22:574. doi: 10.1186/s12859-021-04488-8 (PMC8628452; doi:10.1186/s12859-021-04488-8)
Supplement: Supplementary file 4 — Additional file 4: Figure S3 Exploration of impact of proportionality constant on predicted flux in anaerobic and aerobic in S. cerevisiae: (A) Acetate aerobic (B) Acetate anaerobic (C) Glycerol aerobic (D) Glycerol anaerobic [file 12859_2021_4488_MOESM4_ESM.docx]

**Rintala Dataset**

**A**

**B**


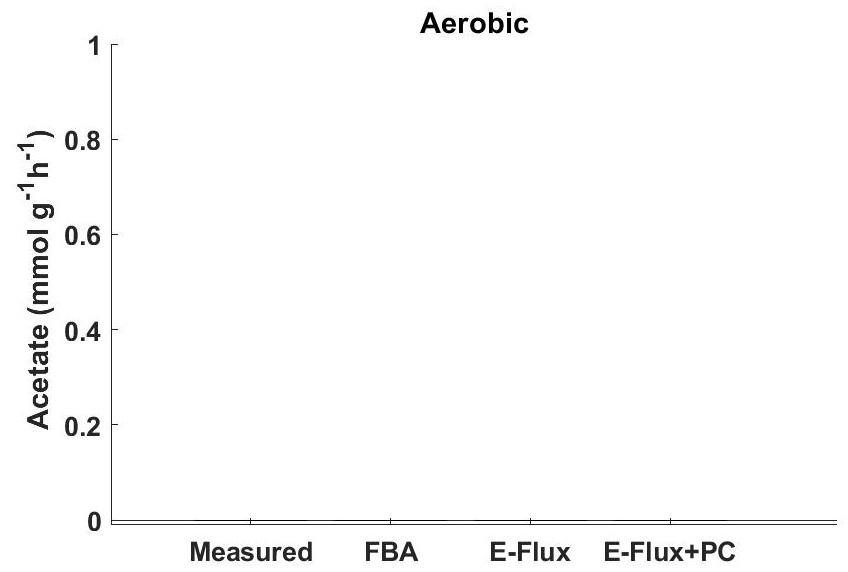
**
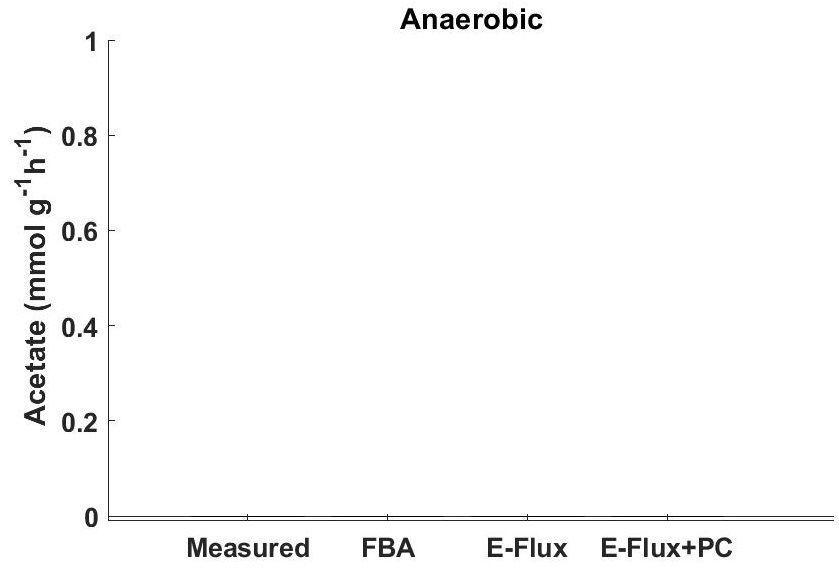
**

**D**

**C**


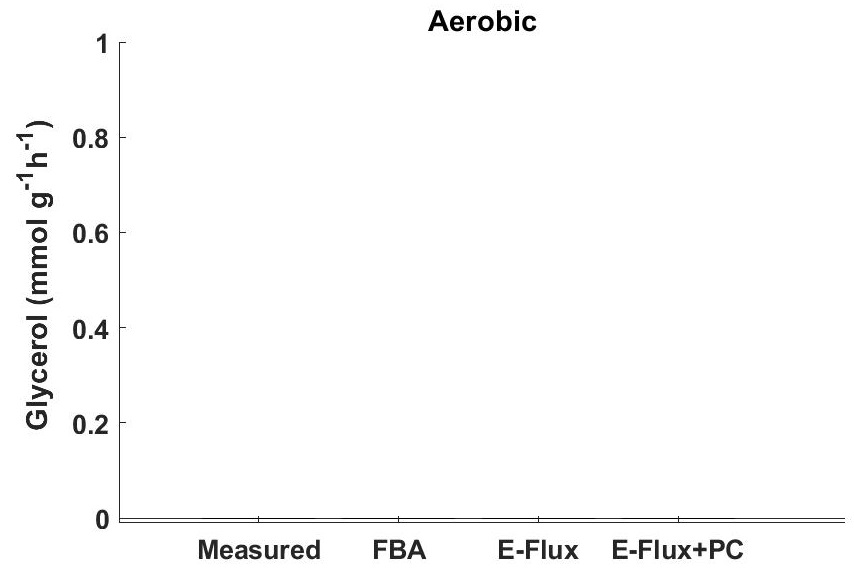


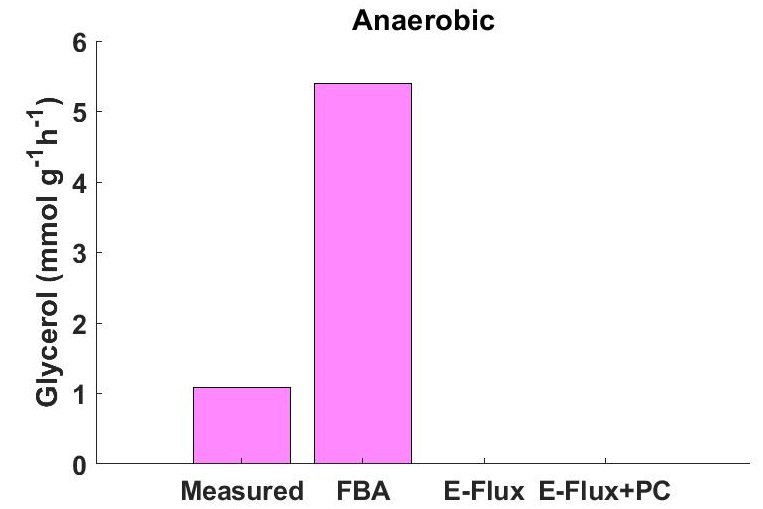


**Additional Figure 3** Exploration of impact of proportionality constant on predicted flux in anaerobic and aerobic in *S. cerevisiae*: (A) Acetate aerobic (B) Acetate anaerobic (C) Glycerol aerobic (D) Glycerol anaerobic
